# Predicting pain reduction following laparoscopic surgery for endometriosis: a retrospective cohort study using UK national and research databases

**DOI:** 10.1136/bmjopen-2025-099374

**Published:** 2025-08-27

**Authors:** Connor Mustard, Kym Snell, Kim May Lee, Cleo Pike, Sharandeep Bhogal, Andrew Horne, Julie Dodds, John Allotey, Carol Rivas, Elizabeth Ball

**Affiliations:** 1 Operational Excellence, Maple, Toronto, Ontario, Canada; 2 Public Health, Epidemiology & Biostatistics, University of Birmingham, Birmingham, UK; 3 King’s College London, London, UK; 4 Barts and the London Pragmatic Clinical Trials Unit, Queen Mary University of London, London, UK; 5 Barts and the London School of Medicine and Dentistry, Queen Mary University of London, London, UK; 6 The University of Edinburgh MRC Centre for Reproductive Health, Edinburgh, UK; 7 Institute of Metabolism and Systems Research and Institute of Applied Health Research, University of Birmingham, Birmingham, UK; 8 Social Research Institute, UCL, London, UK; 9 Centre for Primary Care and Population Health, Queen Mary University of London, London, UK

**Keywords:** Clinical Decision-Making, Triage, EPIDEMIOLOGY, Minimally invasive surgery, GYNAECOLOGY

## Abstract

**Abstract:**

**Objective:**

To develop and validate models to predict which endometriosis patients are likely to experience pain reduction following therapeutic laparoscopy using intraoperative findings and patient characteristics.

**Design:**

A retrospective secondary data analysis with patient workshops.

**Setting:**

Analysis of a UK nationwide, specialist centre, surgical database (British Society for Gynaecological Endoscopy, BSGE) (2013–2019, N=9171) and two research databases, MEDAL (2011–2013, N=667) and LUNA (1998–2005, N=592) for exploratory analyses and external validation.

**Participants:**

Database patients had laparoscopically confirmed (BSGE) or clinically suspected endometriosis (MEDAL, LUNA) and ranged from 16 to 65 years. Patient workshops included UK-wide endometriosis patients from the community, secondary care doctors and endometriosis nurses.

**Primary and secondary outcome measures:**

Following model development and internal validation, primary outcome measures included model performance statistics for discrimination (C-statistic) and calibration (calibration slope and calibration-in-the-large) for pain-improvement models for each of the five clinically meaningful pain domains. Secondary outcome measures included performance statistics for externally validated models and net benefit (using decision curve analysis).

**Results:**

Following internal validation for dyspareunia (pain during sexual intercourse), non-cyclical pelvic pain (NPP), dyschezia (painful defecation) and quality of life our models showed good discrimination ability with C-statistics of 0.768, 0.750, 0.808 and 0.792, respectively. Significant increases in odds of pain relief were associated with trying to conceive for less than 18 months, any treated endometriosis of the ovary or uterosacral ligament or hysterectomy at the time of laparoscopy. For those models for which sufficient data were available to do external validation, dyspareunia and NPP showed good ability to predict pain reduction following surgery with C-statistics of 0.759 and 0.741, respectively, but after external validation only the model for dyspareunia good discriminatory ability (C-statistic=0.718). Despite this, decision curve analysis indicated some net benefit for all externally validated models.

**Conclusions:**

Clinical prediction models can help identify women who will experience pain reduction after therapeutic laparoscopy, but more work is required to externally validate the current models. Removal of ovarian and utero-sacral ligament endometriosis appears to convey pain relief after surgery, whereas removal of superficial peritoneal endometriosis does not.

**Strengths and limitations of this study**

- The main analyses involved development and validation of models using data from a large national surgical database (British Society for Gynaecological Endoscopy) containing data from accredited endometriosis centres, limiting the uncertainty within the models.
- This study benefits from the use of additional datasets to perform external validation of developed models and assess clinical utility using net benefit analysis.
- This study was limited by differences between the model development dataset and external validation datasets regarding which data were captured and the patient populations in the respective databases.

## Introduction

 Endometriosis is a chronic inflammatory condition affecting approximately 6%–10% of women of reproductive age worldwide,^1 2^ defined by the presence of endometrial-like tissue (lesions) outside the uterus. Endometriosis can be associated with debilitating pelvic pain, infertility and fatigue. The pain is often aggravated by menstruation and sexual intercourse and can have devastating effects on quality of life (QoL).

Current gold standard treatment for endometriosis involves a combination of approaches including hormonal treatments (eg, analgesics, drugs that suppress ovarian function), multidisciplinary care (eg, pain management and psychological support) and/or surgery to remove the lesions (usually performed laparoscopically). While there are data to show that 20%–28% of women do not experience pain reduction following surgery,^3 4^ there are currently no clinical models used in routine clinical practice that predict response to therapeutic laparoscopy, and prognosis is often not discussed before surgery.

We previously conducted a systematic review that informed our selection of the location of disease as an important predictor for the regression models, but also reported the current lack of understanding as to why some women do not experience pain reductions following surgery.^5^

The aim of this study was to develop and validate clinical prediction models to help predict clinically meaningful pain reduction following therapeutic laparoscopy.

## Materials and methods

### Study population

Three existing datasets were used for the purposes of this study. The main analysis involved development and validation of predictive models using the British Society for Gynaecological Endoscopy (BSGE) national database. The BSGE maintains a regularly updated UK-wide database containing data from accredited specialist endometriosis centres ensuring surgeries were performed by surgeons with expertise in advanced laparoscopic techniques. For our purposes, women included in the database between January 2013 and December 2019 were analysed. Additionally, the MEDAL study (MRI versus laparoscopy to diagnose the main causes of chronic pelvic pain (CPP) in women; ISRCTN 13028601), and the LUNA randomised controlled trial (Laparoscopic uterosacral nerve ablation for alleviating CPP; ISRCTN 41196151)^6^,^8^ were used for exploratory analyses. The MEDAL study included women who had been referred to a gynaecologist with CPP and had an indication for diagnostic laparoscopy within 6 months, from 26 UK hospitals (December 2011 to September 2013). The LUNA trial included women reporting CPP lasting longer than 6 months who were undergoing diagnostic laparoscopy for differential diagnosis of CPP, from 18 UK hospitals (February 1998 to December 2005). For the datasets listed above, all procedures recorded were conventional therapeutic/diagnostic laparoscopic surgeries. Additionally, there was no risk of patient overlap between datasets as they were collected in separate time periods and through different recruitment channels.

For model development and validation, original datasets were limited based on our inclusion/exclusion criteria. Inclusion criteria: women aged 16–65 years with laparoscopically confirmed (BSGE) or clinically suspected (MEDAL,LUNA) endometriosis, complete baseline and 6-month follow-up pain scores for at least one outcome. Exclusion criteria: absence of endometriosis diagnosis, missing key predictors or missing follow-up data on primary outcomes.

### Study models

For the primary analysis, models were developed using the BSGE dataset, consisting of women who had undergone therapeutic laparoscopies, who had their intraoperative findings (including location of endometriosis) recorded. These findings and other factors of interest were used as candidate factors (hereafter potential predictors) during model development. Following development, all models were internally validated using bootstrap resampling to assess optimism-adjusted performance. External validation of the models, using the MEDAL dataset, was performed as part of our exploratory analyses.

Additional exploratory analyses included developing models using predictors that were known preoperatively. Our aim was to estimate pain reduction in women who had pelvic pain/suspected endometriosis but who had not yet undergone laparoscopy. These additional models were meant to reflect a real-life clinical scenario during preoperative counselling, when only preoperative factors are known. These models were developed using MEDAL and externally validated in LUNA (see online supplemental methods). Analysis of these models was deemed exploratory in nature as these models were likely to be less reliable due to limited development data.

### Outcomes

Five clinically relevant outcomes were chosen to quantify the impact of surgery based on previous work^5^: dysmenorrhoea, dyspareunia, dyschezia, non-cyclical pelvic pain (NPP) and QoL. Pain scores at baseline and follow-up were quantified using a self-reported Visual Analogue Scale (VAS) from 0 to 10. QoL was measured on a barometer for overall health state from 0 to 100 (worst to best QoL). Outcomes were recorded by participants at baseline and at 6 months. Each model was based on achieving a meaningful pain reduction (or improvement in QoL) at 6 months after laparoscopy.

Reported VAS scores were dichotomised to reflect a reduction in pain following surgery. A minimum of 30% reduction from baseline was deemed ‘improved’.^9^ For QoL, a 10-point improvement (out of 100) was chosen according to clinical guidance^10^,^12^ and input from women with endometriosis from the stakeholder workshop.

Based on availability in the datasets used for validation, models were developed, internally validated and then externally validated for dysmenorrhoea, dyspareunia and NPP.

### Predictors

Based on clinical relevance, a list of 12 potential predictors, available in both the BSGE and MEDAL datasets was included for model development. Potential predictors are: baseline pain score, use of hormonal contraception, age, body mass index (BMI), use of any strong painkillers (including analgesics and opiate analgesics), trying to conceive for less than 18 months, any treated ovarian or uterosacral ligament (USL) endometriosis (combined due to small numbers in MEDAL), smoking, any treated obliteration of the cul-de-sac, any treated endometriosis in the peritoneum, any bowel or other treated deep endometriosis (combined due to small numbers in the MEDAL dataset) and hysterectomy at laparoscopy. Potential predictors available in MEDAL (for additional exploratory model development) and LUNA (for external validation of those models) are outlined in the online supplemental methods.

**Figure 1:**
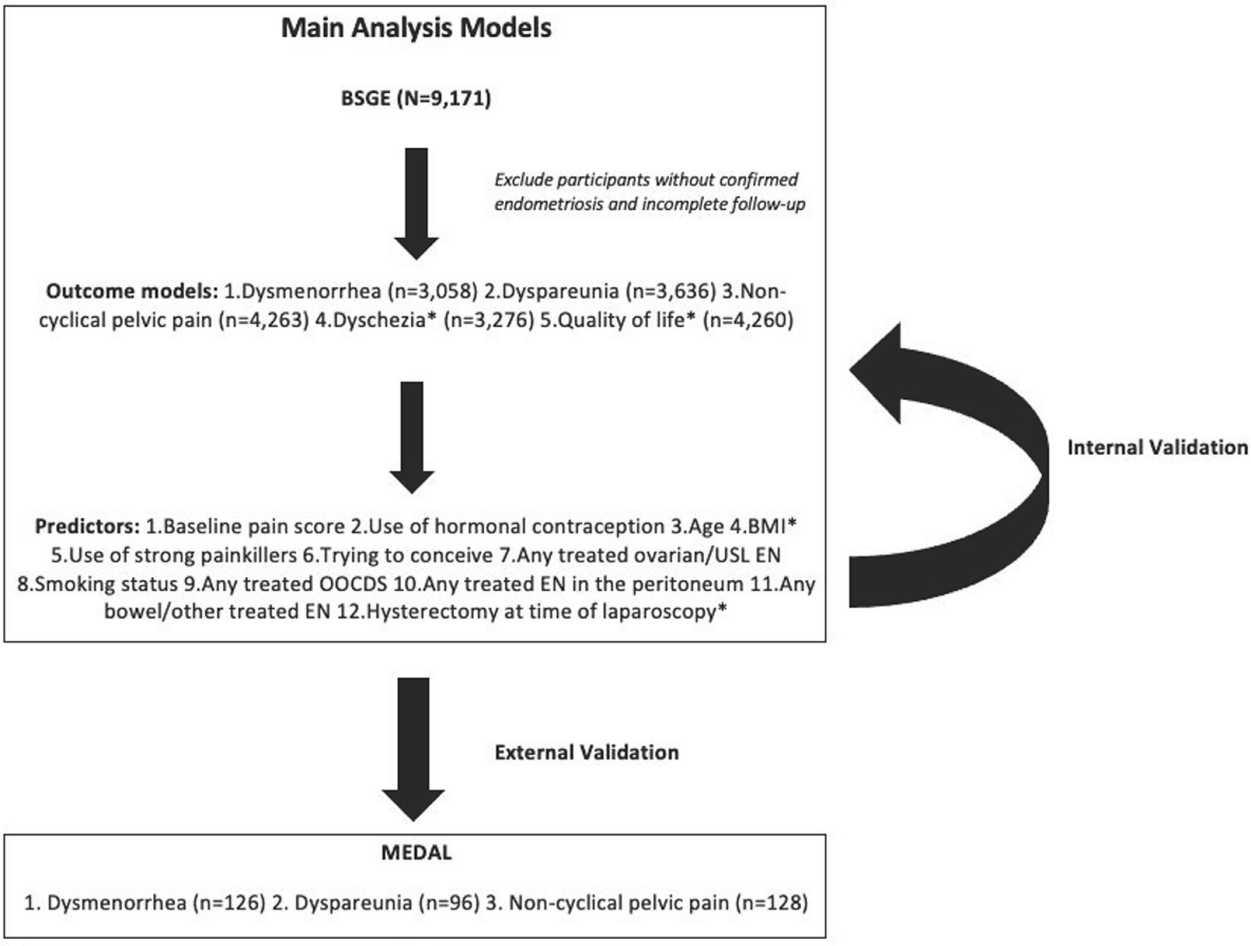
Model development and validation workflow overview. *Outcomes or predictors that were not available for external validation models. BMI, body mass index; BSGE, British Society for Gynaecological Endoscopy; EN, Endometriosis; OOCDS, obliteration of the cul-de-sac.

Fewer outcomes and potential predictors were available in the dataset used for external validation (MEDAL). Two models were developed for each outcome: (1) models using all available predictors in the development data and (2) models limited to those available in external validation data. The outcomes dyschezia and QoL and the predictors hysterectomy at laparoscopy and BMI were not available for external validation. Table 1 shows models developed with all outcomes and all potential predictors (those were only validated internally) and models developed using only those outcomes and predictors available for external validation. Figure 1 presents outcomes, predictors and a workflow for model development and validation for the main analyses (online supplemental figure A1 for additional analyses models).

**Table 1:** Developed model ORs, transformations and performance statistics

	Models for external validation			Models considering full list of candidate factors (internally validated only)				
	Dysmenorhoea model	Dyspareunia model	Non-cyclic pelvic pain model	Dysmenorhea model	Dyspareunia model	Non-cyclic pelvic pain model	Dyschezia model	Quality of life model
Number of obs.	3058	3636	4263	3056	3632	4257	3272	4255
Number of pain change events (%)	1738 (56.8)	1925 (52.9)	2358 (55.3)	1737 (56.8)	1923 (53.0)	2355 (55.3)	1627 (49.7)	2634 (61.9)
Baseline pain score, OR (95% CI)	0.61 (0.54 to 0.69)	0.94 (0.94 to 0.95)	0.93 (0.92 to 0.95)	0.60 (0.54 to 0.68)	0.94 (0.93 to 0.95)	0.94 (0.92 to 0.95)	0.94 (0.93 to 0.95)	0.01 (0.003 to 0.01)
Use of strong painkillers, OR (95% CI)	0.68 (0.57 to 0.81)	0.78 (0.66 to 0.93)	0.86 (0.74 to 1.01)	0.69 (0.57 to 0.82)	0.77 (0.65 to 0.92)	0.86 (0.73 to 1.00)	0.63 (0.52 to 0.76)	0.67 (0.56 to 0.79)
Not trying to conceive vs trying <18 months, OR (95% CI)	1.61 (1.23 to 2.12)	1.41 (1.05 to 1.89)	1.58 (1.20 to 2.09)	1.67 (1.27 to 2.19)	1.51 (1.12 to 2.02)	1.63 (1.23 to 2.16)	1.57 (1.13 to 2.18)	1.78 (1.34 to 2.36)
Not trying to conceive vs trying >18 months, OR (95% CI)	0.97 (0.80 to 1.17)	1.13 (0.92 to 1.38)	1.06 (0.88 to 1.28)	1.00 (0.83 to 1.21)	1.24 (1.01 to 1.53)	1.11 (0.92 to 1.35)	1.12 (0.90 to 1.40)	1.22 (1.00 to 1.49)
Any treated ovarian endometriosis or endometriosis in USL, OR (95% CI)	1.61 (1.20 to 2.16)	1.88 (1.43 to 2.46)	1.76 (1.37 to 2.26)	1.59 (1.19 to 2.14)	1.85 (1.41 to 2.42)	1.68 (1.30 to 2.15)	1.45 (1.05 to 2.01)	1.30 (0.99 to 1.70)
Hysterectomy, OR (95% CI)	No records in MEDAL			1.77 (1.29 to 2.44)	1.70 (1.34 to 2.15)	1.85 (1.51 to 2.26)	1.77 (1.28 to 2.46)	1.57 (1.30 to 1.90)
Age, OR (95% CI)	1.04 (1.02 to 1.05)	1.03 (1.02 to 1.04)	1.04 (1.03 to 1.05)	1.03 (1.02 to 1.04)	1.02 (1.00 to 1.03)	0.01 (0.001 to 0.03)	1.03 (1.01 to 1.04)	–
BMI, OR (95% CI)	Excluded from model build due to missing data			0.98 (0.97 to 1.00)	–	0.99 (0.97 to 1.00)	0.98 (0.96 to 1.00)	0.98 (0.97 to 1.00)
Smoker vs ex-smoker, OR (95% CI)	1.26 (0.96 to 1.67)	1.34 (1.02 to 1.76)	–	1.28 (0.97 to 1.68)	1.32 (1.00 to 1.74)	–	1.17 (0.86 to 1.60)	1.18 (0.90 to 1.55)
Smoker vs never smoker, OR (95% CI)	1.48 (1.19 to 1.85)	1.39 (1.11 to 1.74)	–	1.50 (1.20 to 1.87)	1.40 (1.12 to 1.76)	–	1.33 (1.04 to 1.71)	1.27 (1.02 to 1.59)
Any treated OOCDS, OR (95% CI)	1.48 (1.24 to 1.77)	–	–	1.49 (1.24 to 1.78)	–	–	1.30 (1.08 to 1.56)	1.19 (1.01 to 1.90)
Use of hormonal contraception, OR (95% CI)	1.22 (1.03 to 1.46)	–	–	1.23 (1.03 to 1.46)	–	–	–	–
Any treated endometriosis in the peritoneum, OR (95% CI)	–	–	–	–	–	1.22 (0.97 to 1.55)	–	–
Any treated bowel endometriosis, OR (95% CI)	0.83 (0.68–1.01)	–	–	0.83 (0.68 to 1.02)	–	–	–	–
Intercept (SE)	−0.49 (0.19)	−0.10 (0.17)	0.15 (0.13)	−0.54 (0.19)	−0.21 (0.17)	−0.05 (0.16)	0.02 (0.19)	0.41 (0.17)
Age transformation	Age - 33.77	Age - 35.00	Age - 35.09	Age - 33.76	Age - 34.9879	(Age/10)^-2^–0.08	Age - 34.16	–
BMI transformation	n/a			BMI - 25.64	–	BMI - 26.15	BMI - 25.79	BMI - 26.18
Baseline pain score transformation	((Baseline+1)/10)^–1 ^– 1.10	((Baseline+1)/10)^–2 ^– 2.77	((Baseline+1)/10)^–2 ^– 2.51	((Baseline+1)/10)^–1 ^– 1.10	((Baseline+1)/10)^–2 ^– 2.77	((Baseline+1)/10)^–2 ^– 2.51	((Baseline+1)/10)^–2 ^– 2.67	((Baseline+1)/100)^3 ^– 0.17
Optimism adjusted C-statistic*	0.646	0.759	0.741	0.651	0.768	0.750	0.808	0.792
Optimism adjusted C-slope*	0.934	0.961	0.968	0.920	0.961	0.952	0.952	0.978
Optimism adjusted CITL*	0.000	−0.001	−0.001	0.004	0.005	0.000	−0.002	0.004

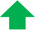
 Predictor may be associated with increased odds of a pain reduction event for the model at 5% significance level (ie, OR>1.0).

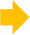
 Predictor does not significantly change odds of a pain reduction event for the model at 5% significance level (ie, OR crosses 1.0).

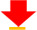
 Predictor may be associated with decreased odds of a pain reduction event for the model at 5% significance level (ie, OR<1.0).

* ORs from originally developed models are presented and represent the odds of a pain reduction event occurring at 6-month follow-up, but performance statistics are presented after internal validation and optimism adjustment of the original models.

OOCDS, obliteration of the cul-de-sac.BMI, body mass index; CITL, calibration-in-the-large; n/a, not available; USL, uterosacral ligament.

### Sample size

Sample size was calculated using the method of Riley *et al*, assuming a Cox-Snell R^2^ of 0.11 (based on prevalence of 50%–60% for the primary outcomes), 12 potential predictors and assuming a shrinkage factor of no less than 0.9 to minimise model overfitting.^13^ Minimum sample size was 824–840. The BSGE database contained complete records, based on selected predictors, for at least 3000 women per outcome, with numbers of pain reduction events ranging from 1627 to 2634 (table 1). Recommendations for external validation are 100–200 outcome events.^14^,^16^ External validation analyses are considered exploratory as MEDAL ranged from 56 to 88 outcome events for the various models.

### Missing data

For pragmatic reasons, a complete-case analysis was used for model development and validation. Predictors missing in 50% or more of observations were excluded from model development as they were not being routinely recorded and would therefore be of limited value in clinical models.

### Statistical analyses

All analyses were performed using Stata V.16.1.

### Model development and internal validation

Logistic regression models were fitted for each outcome, with baseline pain/QoL scores included as mandatory covariates. Predictors were selected using backward elimination with a p<0.157; acting as a proxy for best subset selection using Akaike information criterion.^17^ For continuous predictors, non-linear associations were considered using first-degree fractional polynomial functions (*mfp* command in Stata).

Predictive performance of the models was assessed using measures of discrimination (ability to separate between those with and without an improvement in outcome) and calibration (agreement between observed and predicted probabilities). Discrimination was assessed using the C-statistic, with values that range from 0.5 to 1.0. A value of 1.0 highlights perfect discrimination, while 0.5 highlights the model is no better than chance at predicting improved outcome. Model calibration was assessed using the calibration slope (indicating if models are overfitted or underfitted to the development data, ideal value of 1) and calibration-in-the-large (CITL) which indicates systematic over or underprediction on average (ideal value of 0).

Models were internally validated using bootstrap resampling (with 200 samples) to calculate optimism-adjusted performance statistics.^18^ The optimism-adjusted calibration slope also provides the uniform shrinkage factor, which is used to correct for overfitting. Each final ‘shrunken’ model was produced by multiplying the regression coefficients by the shrinkage factor and re-estimating the intercept.

### External validation and decision curve analysis

Due to limited sample size and population differences in the MEDAL dataset (and more broadly in datasets available at the time of study), external validation and decision curve analysis results could not provide conclusive evidence of external model performance. However, the choice to conduct these analyses was pragmatic, to give indication of possible clinical value of models and to highlight the need for future research.

Models were externally validated using MEDAL and included the same performance statistics listed above.

External validation results are based on models developed with the limited list of predictors. Table 1 presents models that could be externally validated and models that could only be internally validated (ie, containing the full list of potential predictors). To assess the potential clinical utility of the externally validated models, we performed decision curve analysis using net benefit.^19 20^ The net benefit of a model is calculated and plotted across a range of threshold probabilities—in our study—of a pain reduction event and compared with the net benefit of strategies of treating all women or none with laparoscopic surgery. The treatment method with the highest ‘net benefit’ over the clinically relevant threshold probabilities is deemed to be the method with the greatest clinical utility.^20^

### Stakeholder workshops

Two facilitated mixed stakeholder workshop discussions were held with service users (before the study) and healthcare professionals and service users (when results were available). Participants were recruited by opportunistic convenience sampling (n=10) women with lived experience of endometriosis, doctors and nurses were approached through existing networks and social media. Workshops were held virtually. Women, who had their identity disguised, were videoed for the codesign touchpoint films, which served as a starting point for discussion. Participants were asked to focus on key touchpoints, that is, points within the endometriosis pathway where our clinical prediction model might have an emotional or clinical impact or where there may be impediments to its sustainable implementation.

Workshops lasted approximately 2 hours. In the second workshop, we showed attendees a rough simulation of what the model would do. This enabled attending clinicians to comment on its usefulness and how they would like to access it. We also asked participants what a meaningful minimal change in pain score after surgery is and how it should be reported in the model. Data were analysed using a rapid thematic analysis approach. Given that we were aiming for information on operationalisation of the aid, which was descriptive, we did not undertake a more conceptual thematic analysis but rather clustered the data into topics of relevance from the transcripts.

## Results

### Study population

Online supplemental table A1 presents patient characteristics from the original datasets and online supplemental table A2 highlights demographic characteristics for each dataset for women with confirmed (BSGE) or suspected (MEDAL/LUNA) endometriosis and complete follow-up for pain outcomes of interest. Online supplemental table A3 presents a descriptive summary for model development (BSGE) and external validation (MEDAL) datasets, for each available pain outcome and includes demographic characteristics and potential candidate factors. Online supplemental table A4 presents a descriptive summary for exploratory model development (MEDAL) and external validation (LUNA) datasets. Age of women in BSGE models (range: 33.8–35.2 years) was higher than MEDAL (range: 30.9–31.1 years). A number of records and pain change events for model development are highlighted in table 1.

### Model performance

ORs for predictors represent increased (OR>1.0) or decreased (OR<1.0) odds of a pain reduction event, associated with that predictor. Predictive performance statistics describe model calibration and how well models predict women who would experience a pain reduction vs those who would not, after internal validation. Table 1 presents ORs, 95% CIs, transformations and performance statistics for each model. Additionally, table 1 presents how predictors contribute to odds of a pain reduction event (at 5% significance level).

Trying to conceive for less than 18 months and any treated ovarian endometriosis or treated endometriosis in the USL were found to be significantly associated with the outcome, increased odds of a pain reduction following treatment, as they were selected in all the developed models (table 1). Increasing age was selected in all externally validated models and hysterectomy at time of laparoscopy was selected in each internally validated only model (table 1). Surgical treatment of peritoneal endometriosis was not retained during model development, in any of the three externally validated models (table 1).

For models developed for internal validation only (ie, using full list of predictors and outcomes) the dyspareunia, NPP, dyschezia and QoL models showed acceptable to good discrimination ability with C-statistics of 0.768, 0.750, 0.808 and 0.792, respectively (table 1). Additionally, table 1 highlights that all five internally validated models showed little evidence of model overfitting with calibration slopes all >0.920.

For models with the reduced list of potential predictors, part of the exploratory external validation, dyspareunia and NPP models showed an acceptable to good ability to separate women who did and did not experience a pain reduction event (C-statistics 0.759 and 0.741, respectively) (table 1). The dysmenorrhoea model had poorer discrimination ability (C-statistic=0.646) (table 1). On internal validation, calibration slopes ranged from 0.934 to 0.968, suggesting minimal overfitting (table 1).

**Figure 2:**
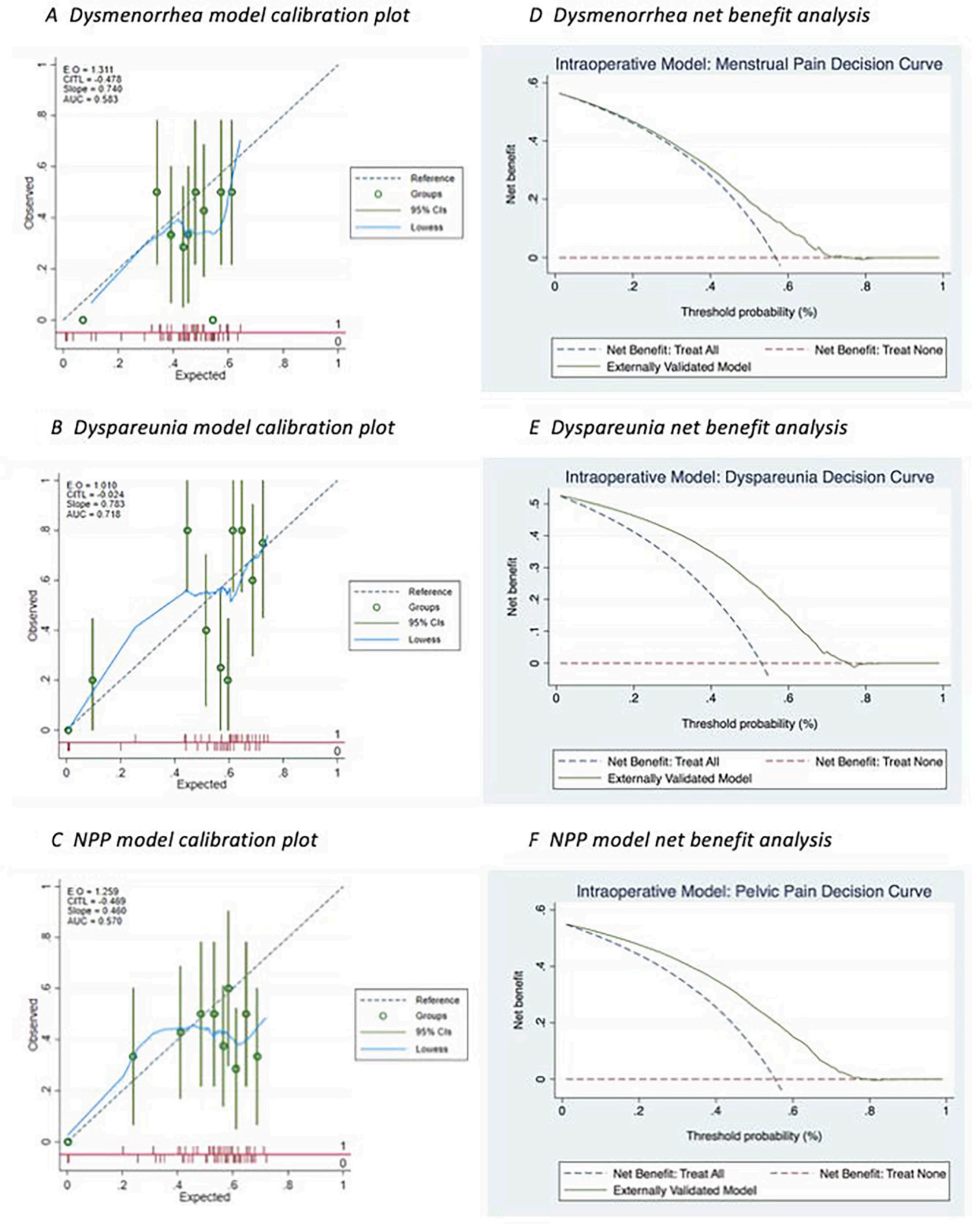
External validation (in MEDAL) calibration plots and net benefit analysis for models developed in BSGE. AUC, Area Under the Receiver Operating Characteristic curve; BSGE, British Society for Gynaecological Endoscopy; CITL, calibration-in-the-large; NPP, non-cyclical pelvic pain.

Figure 2 shows performance statistics following external validation in MEDAL of the three developed models. The dyspareunia model showed acceptable to good discrimination (C-statistic=0.718) and the CITL was close to zero (figure 2B). Discrimination ability for the dysmenorrhoea (C-statistic=0.583) and NPP (C-statistic=0.570) models indicated they were only slightly better than chance at predicting pain reduction events (figure 2A,C). Furthermore, the calibration plots show they predicted poorly when applied to women in MEDAL and CITL values indicate they systematically overpredict pain change events. Dyspareunia and NPP models showed positive net benefit when compared with treating all women or none for thresholds between 0 and 0.80 (figure 2E,F).

Additional analyses models (ie, models developed in MEDAL and externally validated in LUNA) showed poorer predictive ability for both the internally validated only models (C-statistics ranging from 0.631 to 0.719) (online supplemental table A5) and the models for external validation (C-statistics 0.610 and 0.677) (online supplemental table A5). Following external validation in LUNA, the models, like those in the main analysis, hardly performed better than chance at predicting pain reduction events (online supplemental figure A2). Both models showed some net benefit (online supplemental figure A2). Full presentation of results for the additional analyses can be found in the online supplemental results section.

### Qualitative data

Workshop participants were recruited by opportunistic convenience sampling. Women with lived experience of endometriosis, doctors and endometriosis specialist nurses were approached through existing networks and social media. To gather feedback about the research idea before the study, we held a face-to-face focus meeting with nine women in June 2017. After that, the workshops were held; a workshop in July 2019 was held with five women, of which two had their identity disguised and were videoed for the codesign touchpoint films, which served as a starting point for discussion. The second workshop, presenting the final models, was held on September 2020 with three doctors, two endometriosis nurses and four women.

Workshop participants agreed the tool would focus clinic discussions and help women manage expectations about surgery and the likely prognosis. Some clinicians had not previously routinised prognostic discussions into their clinical practice. All agreed that accuracy, credibility, reliability and predictive value were important to establish before using the tool routinely. Women desired the same access to the tool as healthcare professionals, giving a sense of empowerment. Stakeholders agreed signposts to alternative treatments were important for women to make an informed choice. Women felt 30% pain reduction was clinically meaningful, but some women would go ahead with surgery even with lower predicted pain relief, and it was argued surgery should not be denied to them. The tool was seen as an aid to discussion rather than something the clinician alone used to triage for or against surgery.

## Discussion

### Main findings

To our knowledge, there are currently no models which predict pain relief after therapeutic laparoscopy in women with endometriosis. In this study, we have shown that it is possible to develop models that predict the probability of improvement in pain and QoL outcomes based on patient background characteristics and location of endometriosis. In predictive modelling, C-statistics between 0.7 and 0.8 are generally interpreted as demonstrating acceptable to good discrimination, indicating that the models can distinguish between patients with and without clinically meaningful pain reductions. Thus, most of the regression models displayed acceptable to good discriminative ability and relatively low levels of overfitting on internal validation but performed relatively poorly in the external datasets. However, decision curve analysis for each of the externally validated models indicated there was still some net benefit. Furthermore, age (*all but one model), trying to conceive for less than 18 months (vs not trying), hysterectomy at time of laparoscopy and treated ovarian or USL endometriosis were selected in each model indicating they may be strong predictors of pain reduction following surgery. In contrast, superficial peritoneal endometriosis was a poor predictor of postoperative pain relief in our study and was only included in one model. ‘Use of strong painkillers’ and ‘increasing baseline pain scores’ were selected in each of the models and were associated with a decreased odds of pain reduction. This relationship is in line with observations on response to treatment seen for other chronic pain conditions.^21^,^24^

Clinically, the impact of hysterectomy on pain reduction can be explained by the removal of concurrent adenomyosis, a common comorbidity involving the uterine wall in endometriosis patients. An inverse relationship of higher pain scores associated with a higher pain reduction in women with stage 4 endometriosis (as compared with lower stages) has been previously reported.^25^ However, these authors did not state endometriosis location. Other reports^426^,^28^ suggest pain reduction events may be more closely associated with higher stage endometriosis in specific locations as opposed to an individual’s pain severity prior to surgery.^5^ In fact, the current findings support that location of endometriosis is key: Treated ovarian or USL endometriosis was selected in all developed models as a significant predictor of pain reduction. This is in line with the systematic review findings that removal of deep uterosacral endometriosis reduced all five pain types when women were treated in specialist centres. Uterosacral and ovarian endometriosis (which can both be detected preoperatively) have been reported previously as correlated to the severity of pelvic pain scores before surgery.^29^ The present work indicates that removing this pathology reduces pain scores postoperatively. Conversely, treatment of superficial peritoneal endometriosis (usually stages 1–2) was only selected as a predictor in one of the models. This is in keeping with previous findings from our systematic review,^5^ indicating treatment of stage I–II endometriosis showed minimal or no significant pain relief after treatment. This is under investigation in a multicentre randomised controlled trial (ESPRIT2 IRAS 291525).

### Strengths

The inclusion (or exclusion) of certain predictors in the models, highlighting them as potentially meaningful (or not), is strengthened by the size of the datasets used for model development. Models developed for the main analysis (using BSGE) were at least three times the size of the calculated minimum sample size (824–840), indicating that selected predictors are less likely to be influenced by uncertainty within the models.^13^ Additionally, despite the differences between the datasets, this study benefits from the use of additional datasets (MEDAL and LUNA) to perform additional exploratory analyses. External validation of the models developed using BSGE allows for comment on the clinical utility of the developed models using net benefit analysis. Furthermore, the additional analyses allow us to explore the performance and clinical utility of models developed using only predictors known prior to surgery, reflecting a pragmatic clinical setting. These models cannot provide conclusive evidence of model performance, but they can give an indication of the possible value of the models and inspire further research/updating of datasets.

### Limitations

We acknowledge limitations to this study: First, complete case analysis was considered when preparing datasets for model development. The MEDAL and LUNA publications did not suggest their findings were sensitive to the presence of missing values and multiple imputation was deemed beyond the scope of this project. Second, differences in surgical expertise may play a role in this study. While intersurgeon variability cannot be ruled out, our use of data from BSGE-accredited centres helps minimise variability in surgical expertise, though this variability may remain as a potential unmeasured confounder. Additionally, datasets for model development and external validation were very different, particularly for the models developed in BSGE and externally validated in MEDAL. BSGE was composed almost exclusively of women suffering from severe endometriosis, while LUNA and MEDAL contained women with endometriosis (of varying stage) and without endometriosis, which may explain in part why developed models did not perform as well in the external validation datasets. However, the use of MEDAL and LUNA datasets in this study was pragmatic (rather than guided by the best possible option) as these were the datasets available at the time. Furthermore, differences in data available in the datasets themselves limited predictors and reduced the number of models that could be externally validated. The small sample size of MEDAL and LUNA limited the validity of the investigation, particularly due to low event numbers in MEDAL for external validation and additional model development. Therefore, there is likely to be large uncertainty in model performance measures using these datasets. In future work, we recommend using larger, more comparable datasets when validating.

### Interpretation

The decision curve analyses, despite being exploratory, show some net benefit of the models. Further interpretation of the curves is difficult as we are not suggesting that prediction models directly inform the decision to treat surgically (eg, if an individual’s predicted probability is greater than a specified threshold), but rather that such models can aid the discussion between patient and clinician as part of the decision-making process. Nevertheless, seeing some net benefit in each of the externally validated models is encouraging and suggests with further work, prediction models for treatment success can be useful for counselling patients. However, during preoperative patient counselling, intraoperative findings are usually not known. Therefore, future work may incorporate surrogate markers for intraoperative findings of interest, such as uterosacral and ovarian endometriosis, into preoperative models which will make them more usable in clinical decision-making for or against surgery.

## Conclusions

Previous authors^30 31^ have aimed to create tools to predict the diagnosis of any endometriosis or deep infiltrating endometriosis, respectively, based on symptoms and participant characteristics. The immediate impact of our study is the development and validation of models to predict the probability of pain relief for the most important types of endometriosis-associated pain. We have provided proof of concept that it is possible to develop such models and that they can provide some net benefit. Additionally, the finding that treated ovarian/USL endometriosis was a significant predictor of pain reduction in each of the developed models (supporting findings from our previous systematic review) may be highly relevant to clinical care, as uterosacral endometriosis can be determined prior to surgery, by pelvic examination and assessment and endometrioma by standard ultrasound scanning.

To our knowledge, our work to develop clinical prediction models to identify women who may benefit from laparoscopic surgery to treat endometriosis is unique. We developed models that, after internal validation, performed well and displayed a good ability to predict women who may benefit from treatment. If we combine these models with larger/more comparable datasets for external validation, this could improve the observed net benefit of the models and may greatly help patients suffering from endometriosis. In our study, we report overall net benefit of the externally validated models and highlight potential predictors of surgical success (such as any treated ovarian/USL endometriosis). Our models have the potential to be used in tandem with other clinical decision tools^30 31^, particularly as we learn more about predictors associated with increased odds of a pain reduction event.^30 31^

## Supplementary material

10.1136/bmjopen-2025-099374 online supplemental file 1

## Data Availability

Data are available on reasonable request.
