# Supplementary material for: Predicting pain reduction following laparoscopic surgery for endometriosis: a retrospective cohort study using UK national and research databases
Source: BMJ Open. 2025 Aug 27;15(8):e099374. doi: 10.1136/bmjopen-2025-099374 (PMC12410623; doi:10.1136/bmjopen-2025-099374)
Supplement: online supplemental file 1 [file bmjopen-15-8-s001.docx]

**Supplementary Materials**

***Supplementary Methods***

*Outcomes and Predictors*

Models used for additional analyses were developed, internally validated, and externally validated for dysmenorrhea and dyspareunia only. For these models, nine potential predictors were available in both the MEDAL and LUNA datasets. Potential predictors included: baseline pain score, use of hormonal contraception, age, use of strong painkillers, number of live births (no live births, one, two or more), alcohol use, blood in stool, having two or more miscarriage/stillbirths, and depression/anxiety. As in the models developed and validated using BSGE, two models were developed for each outcome for our exploratory analyses. NPP, Dyschezia, and QoL outcomes and alcohol use, blood in stool, and having two or more miscarriage/stillbirths were not available for externally validated models. Table A3 presents a descriptive summary for model development (MEDAL) and external validation (LUNA) datasets, for each available pain outcome and includes demographic characteristics and potential candidate factors. Figure A1 outlines a workflow for models used for additional analyses.

*Sample size*

Datasets for additional analyses were developed in much smaller datasets thus more overfitting and more shrinkage is expected to be necessary. LUNA had 119 and 139 pain reduction events for external validation.

*Model development and validation*

Models for additional analyses were developed, internally validated, and externally validated in the same manner as the primary analyses. Additionally, net benefit was assessed using decision curves as described in the main text.

***Supplementary Results***

*Study population*

Mean age of women in LUNA was substantially lower than BSGE (35.1) and MEDAL (35.0) at 30.9 (SD 7.2) years. Similar to MEDAL (85%), 84% of women in the LUNA dataset reported being sexually active (Table A2).

*Model performance – Additional analyses models*

Additional analyses model results are presented in Table A4. Table A4 highlights odds ratios, predictive performance statistics, and pictorial presentation of data in the same manner as the analyses in the main text.

For models developed for internal validation only (i.e. using full list of predictors and outcomes) each of the three models, dysmennorhea, dyspareunia, and NPP, displayed moderate predictive ability with C-statistics of 0.631, 0.705, and 0.719 respectively. More model overfitting was expected with calibration slopes ranging from 0.610 to 0.731 (Table A4).

For models developed for external validation, the dyspareunia model displayed moderate predictive ability (optimism-adjusted C-statistic = 0.677) (Table A4). Calibration slopes for the dysmenorrhea and dyspareunia models were 0.700 and 0.763 respectively, indicating considerable model overfitting (Table A4).

Figure A2 shows performance statistics following external validation of the two developed dysmenorrhea and dyspareunia models. Discrimination was poor with optimism-adjusted C-statistics of 0.562 and 0.593 respectively (Figure A2). The dysmenorrhea model’s calibration slope (0.268) suggests the model predicts poorly overall for women in LUNA (Figure A2). The calibration slope, CITL, and the calibration plot for the dyspareunia model shows there was less agreement between the observed and expected outcomes when validated in LUNA. Predicted probabilities were consistently higher than the observed probabilities for women in LUNA.

Decision curve analysis for the models showed positive net benefit that surpasses treating or not treating all women at threshold probabilities greater than 10% (Figure A2-C & A2-D).


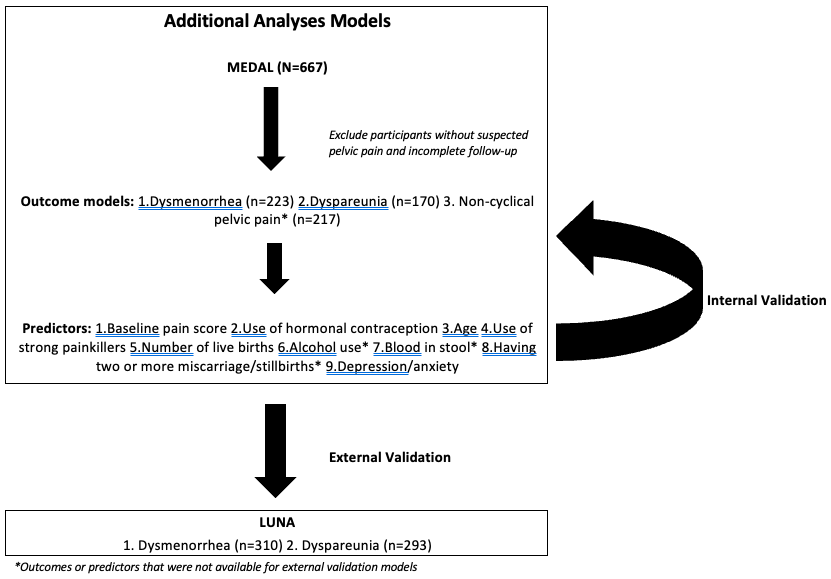


Figure A1: Additional analyses – Model development and validation workflow overview

**
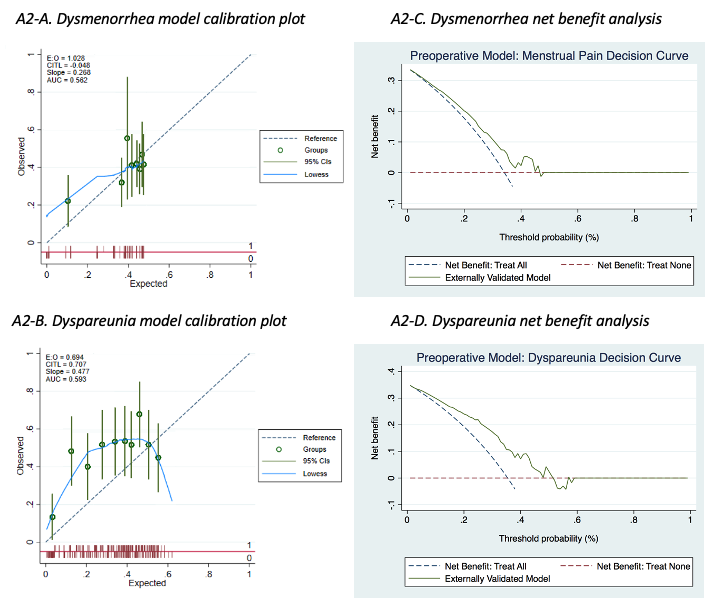
**

Figure A2: Additional analyses – External validation (in LUNA) calibration plots and net benefit analysis for models developed in MEDAL

**Supplementary Tables**

*Table A1: Original study dataset populations and characteristics*

| **Database** | **Study dates** | **Eligible participant number and age range** | **Participant characteristics or inclusion/exclusion** |
| --- | --- | --- | --- |
| BSGE | January 2013 – December 2019 | 9,171 participants aged 17-63 | Women receiving laparoscopic treatment for confirmed endometriosis in specialist centres in the UK |
| MEDAL | December 2011 – September 2013 | 667 participants aged 16-65 | Women with pelvic pain (including suspected endometriosis) participating in a multicentre trial. Women were excluded if they were pregnant, had a hysterectomy, or a previously established cause of CPP. |
| LUNA | February 1998 – December 2005 | 592 participants aged 17-64 | Women from 18 UK hospitals with CPP for at least six months (including suspected endometriosis), with a planned diagnostic laparoscopy were eligible. Women with previous laser ablation or previous endometriosis surgery, confirmed moderate or severe endometriosis or hysterectomy were excluded. |

| **Table A2: Demographic characteristics of study datasets for women with confirmed (BSGE) or suspected (MEDAL / LUNA) endometriosis and complete follow-up for pain outcomes** | | | |
| --- | --- | --- | --- |
|  | **BSGE dataset** | **MEDAL dataset** | **LUNA dataset** |
| **DEMOGRAPHIC CHARATERISTICS** |  |  |  |
| **Number of women** | 4,263 | 223 | 310 |
| **Age** mean (SD) | 35.1 (7.4) | 35.0 (7.2) | 30.9 (7.2) |
| missing, n (%) | 0 | 0 | 0 |
| **Height (m)** mean (SD) | 1.6 | 1.6 (0.1) | - |
| missing, n (%) | 6 (0.1) | 72 (32.3) |  |
| **Weight (kg)** mean (SD) | 69.9 (14.8) | 65.0 (10.6) | - |
| missing, n (%) | 0 | 117 (52.4) |  |
| **BMI** mean (SD) | 26.1 (5.5) | 24.7 (4.7) | - |
| missing, n (%) | 6 (0.1) | 181 (81.2) |  |
| **Smoking, N (%)** |  |  |  |
| Current Smoker | 535 (12.5) | 46 (20.6) | - |
| Ex-smoker | 728 (17.1) | 51 (22.9) | - |
| Never smoked | 3000 (70.4) | 120 (53.8) | - |
| Missing | 0 | 6 (2.7) | - |
| **Sexually active, N (%)** |  |  |  |
| Yes | - | 189 (84.8) | 259 (83.6) |
| No | - | 32 (14.4) | 46 (14.8) |
| Missing | - | 2 (0.9) | 5 (1.6) |
| **Having Periods, N (%)** |  |  |  |
| Yes | - | 211 (94.6) | - |
| No | - | 12 (5.4) | - |
| Missing | - | 0 | - |

| **Table A3: Descriptive summary for model development and external validation datasets** | | | | | | | | |
| --- | --- | --- | --- | --- | --- | --- | --- | --- |
|  | **Development/Validation Complete Datasets** | | | | | **External Validation Complete Datasets** | | |
| **DEMOGRAPHIC CHARACTERISTICS** | **BSGE Dysmenorrhea model** | **BSGE Dyspareunia model** | **BSGE Non-cyclic pain model** | **BSGE Dyschezia model** | **BSGE QoL model** | **MEDAL Dysmenorrhea model** | **MEDAL Dyspareunia model** | **MEDAL Non-cyclic pain model** |
| **Number of women** | 3,058 | 3,636 | 4,263 | 3,276 | 4,260 | 126 | 96 | 128 |
| **Age** |  |  |  |  |  |  |  |  |
| Mean (SD) | 33.77 (6.85) | 34.99 (7.21) | 35.09 (7.38) | 34.17 (6.95) | 35.22 (7.40) | 31.06 (5.95) | 30.92 (6.22) | 31.05 (5.91) |
| **Height** |  |  |  |  |  |  |  |  |
| Mean (SD) | 1.63 (0.07) | 1.64 (0.07) | 1.64 (0.07) | 1.64 (0.07) | 1.64 (0.07) | 1.63 (0.07) | 1.62 (0.07) | 1.63 (0.07) |
| **Weight** |  |  |  |  |  |  |  |  |
| Mean (SD) | 68.55 (14.34) | 69.75 (14.45) | 69.89 (14.77) | 68.96 (14.50) | 70.00 (14.78) | 65.33 (12.46) | 63.99 (9.92) | 65.33 (12.46) |
| **Smoking, N (%)** |  |  |  |  |  |  |  |  |
| Current Smoker | 409 (13.37) | 467 (12.86) | 535 (12.55) | 421 (12.85) | 524 (12.30) | 18 (14.29) | 14 (14.58) | 18 (14.06) |
| Ex-smoker | 474 (15.50) | 639 (17.57) | 728 (17.08) | 507 (15.48) | 725 (17.02) | 28 (22.22) | 22 (22.92) | 28 (21.88) |
| Never smoked | 2,175 (71.12) | 2,530 (69.58) | 3,000 (70.37) | 2,348 (71.67) | 3,011 (70.68) | 80 (63.49) | 60 (62.50) | 82 (64.06) |
| **BMI** |  |  |  |  |  |  |  |  |
| Mean (SD) | 25.64 (5.37) | 26.09 (5.40) | 26.15 (5.48) | 25.79 (5.40) | 26.18 (5.49) | - | - | - |
| **Still sexually active, N (%)** |  |  |  |  |  |  |  |  |
| Yes | - | - | - | - | - | 126 (100) | 96 (100) | 128 (100) |
| No | - | - | - | - | - | 0 | 0 | 0 |
| **Still having Periods, N (%)** |  |  |  |  |  |  |  |  |
| Yes | - | - | - | - | - | 122 (96.83) | 90 (93.75) | 124 (96.88) |
| No | - | - | - | - | - | 4 (3.17) | 6 (6.25) | 4 (3.13) |
|  |  |  |  |  |  |  |  |  |
| **Potential candidate factors** | | | | | | | | |
| **Age** |  |  |  |  |  |  |  |  |
| Mean (SD) | 33.77 (6.85) | 34.99 (7.21) | 35.09 (7.38) | 34.17 (6.95) | 35.22 (7.40) | 31.06 (5.95) | 30.92 (6.22) | 31.05 (5.91) |
| **Smoking, N (%)** |  |  |  |  |  |  |  |  |
| Current Smoker | 409 (13.37) | 467 (12.86) | 535 (12.55) | 421 (12.85) | 524 (12.30) | 18 (14.29) | 14 (14.58) | 18 (14.06) |
| Ex-smoker | 474 (15.50) | 639 (17.57) | 728 (17.08) | 507 (15.48) | 725 (17.02) | 28 (22.22) | 22 (22.92) | 28 (21.88) |
| Never smoked | 2,175 (71.12) | 2,530 (69.58) | 3,000 (70.37) | 2,348 (71.67) | 3,011 (70.68) | 80 (63.49) | 60 (62.50) | 82 (64.06) |
| **Contraception, N (%)** |  |  |  |  |  |  |  |  |
| Yes | 848 (27.73) | 1,046 (28.77) | 1,268 (29.74) | 925 (28.24) | 1,254 (29.44) | 80 (63.49) | 64 (66.67) | 82 (64.06) |
| No | 2,210 (72.27) | 2,590 (71.23) | 2,995 (70.26) | 2,351 (71.76) | 3,006 (70.56) | 46 (36.51) | 32 (33.33) | 46 (35.94) |
| **Trying for baby (>18 mths), N (%)** |  |  |  |  |  |  |  |  |
| No | 2,049 (67.00) | 2,551 (70.16) | 3,132 (73.47) | 2,261 (69.02) | 3,124 (73.33) | 102 (80.95) | 72 (75.00) | 102 (79.69) |
| Yes, but less than 18 months | 300 (9.81) | 329 (9.05) | 348 (8.16) | 307 (9.73) | 353 (8.29) | 8 (6.35) | 4 (4.17) | 8 (6.25) |
| Yes, more than 18 months | 709 (23.19) | 756 (20.79) | 783 (18.37) | 708 (21.61) | 783 (18.38) | 16 (12.70) | 20 (20.83) | 18 (14.06) |
| **Strong Pain Killer, N (%)** |  |  |  |  |  |  |  |  |
| Yes | 737 (24.10) | 918 (25.25) | 1,119 (26.25) | 799 (24.39) | 1,107 (25.99) | 100 (79.37) | 78 (81.25) | 102 (79.69) |
| No | 2,321 (75.90) | 2,718 (74.75) | 3,144 (73.75) | 2,477 (75.61) | 3,153 (74.01) | 26 (20.63) | 18 (18.75) | 26 (20.31) |
| **Inter-operative factors** |  |  |  |  |  |  |  |  |
| **Location Ovary /USL, N (%)** |  |  |  |  |  |  |  |  |
| Yes | 2,836 (92.74) | 3,353 (92.22) | 3,933 (92.26) | 3,037 (92.70) | 3,932 (92.30) | 90 (71.43) | 70 (72.92) | 92 (71.88) |
| No | 222 (7.26) | 283 (7.78) | 330 (7.74) | 239 (7.30) | 328 (7.70) | 36 (28.57) | 26 (27.08) | 36 (28.13) |
| **Location OOCDS/cul de sac, N (%)** |  |  |  |  |  |  |  |  |
| Yes | 1,951 (63.80) | 2,376 (65.35) | 2,810 (65.92) | 2,102 (64.16) | 2,849 (66.88) | 18 (14.29) | 14 (14.58) | 20 (15.63) |
| No | 1,107 (36.20) | 1,260 (34.65) | 1,453 (34.08) | 1,174 (35.84) | 1,411 (33.12) | 108 (85.71) | 82 (85.42) | 108 (84.38) |
| **Location Bowel/bladder, N (%)** |  |  |  |  |  |  |  |  |
| Yes | 2,374 (77.63) | 2,888 (79.43) | 3,385 (79.40) | 2,544 (77.66) | 3,413 (80.12) | 14 (11.11) | 10 (10.42) | 16 (12.50) |
| No | 684 (22.37) | 748 (20.57) | 878 (20.60) | 732 (22.34) | 847 (19.88) | 112 (88.89) | 86 (89.58) | 112 (87.50) |
| **Location Peritoneum, N (%)** |  |  |  |  |  |  |  |  |
| Yes | 2,762 (90.32) | 3,265 (89.80) | 3,831 (89.87) | 2,961 (90.38) | 3,814 (89.53) | 110 (87.30) | 86 (89.58) | 112 (87.50) |
| No | 296 (9.68) | 371 (10.20) | 432 (10.13) | 315 (9.62) | 446 (10.47) | 16 (12.70) | 10 (10.42) | 16 (12.50) |
| **BMI** |  |  |  |  |  |  |  |  |
| Mean (SD) | 25.64 (5.37) | 26.09 (5.40) | 26.15 (5.48) | 25.79 (5.40) | 26.18 (5.49) | - | - | - |
| **Hysterectomy, N (%)** |  |  |  |  |  |  |  |  |
| Yes | 257 (8.40) | 761 (20.93) | 921 (21.60) | 398 (12.15) | 961 (22.56) | - | - | - |
| No | 2,801 (91.60) | 2,875 (79.07) | 3,342 (78.40) | 2,878 (87.85) | 3,299 (77.44) | - | - | - |
| **Model baseline pain score, Mean (SD)** | 8.11 (2.33) | 5.01 (3.38) | 5.32 (3.06) | 5.12 (3.71) | 54.55 (24.40) | 7.00 (2.91) | 5.52 (3.30) | 4.75 (2.91) |

| **Table A4: Additional analyses - Descriptive summary for model development and external validation datasets** | | | | | |
| --- | --- | --- | --- | --- | --- |
|  | **Development/Validation Complete Datasets** | | | **External Validation Comlete Datasets** | |
| **DEMOGRAPHIC CHARATERISTICS** | **MEDAL Dysmenorrhea model** | **MEDAL Dyspareunia model** | **MEDAL Non-cyclical pain model** | **LUNA Dysmenorrhea model** | **LUNA Dyspareunia model** |
| **Number of women** | 223 | 170 | 217 | 310 | 293 |
| **Age** |  |  |  |  |  |
| Mean (SD) | 34.98 (7.19) | 34.41 (6.63) | 34.77 (7.16) | 30.87 (7.23) | 30.57 (7.28) |
| **Still sexually active, N (%)** |  |  |  |  |  |
| Yes | 189 (84.75) | 166 (97.65) | 185 (85.25) | 259 (83.55) | 265 (90.44) |
| No | 32 (14.35) | 4 (2.35) | 0 | 46 (14.84) | 24 (8.19) |
| **Still having Periods, N (%)** |  |  |  |  |  |
| Yes | 211 (94.62) | 144 (84.71) | 205 (94.47) | - | - |
| No | 12 (5.38) | 26 (15.29) | 12 (5.53) | - | - |
| **Height** |  |  |  |  |  |
| Mean (SD) | 1.62 (0.06) | 1.61 (0.06) | 1.62 (0.06) | - | - |
| **Weight** |  |  |  |  |  |
| Mean (SD) | 65.04 (10.64) | 68.54 (11.44) | 65.16 (10.82) | - | - |
| **Smoking, N(%)** |  |  |  |  |  |
| Current Smoker | 46 (20.63) | 36 (21.18) | 46 (21.20) | - | - |
| Ex-smoker | 51 (22.87) | 44 (25.88) | 49 (22.58) | - | - |
| Never smoked | 120 (53.81) | 88 (51.76) | 116 (53.46) | - | - |
| **Marital Status, N (%)** |  |  |  |  |  |
| Single | - | - | - | 107 (34.52) | 85 (29.01) |
| Married | - | - | - | 116 (37.42) | 123 (41.98) |
| Divorced | - | - | - | 9 (2.90) | 7 (2.39) |
| Living with partner | - | - | - | 73 (23.55) | 74 (25.26) |
| **Potential candidate factors** | | | | | |
| **Age** |  |  |  |  |  |
| Mean (SD) | 34.98 (7.19) | 34.41 (6.63) | 34.77 (7.16) | 30.87 (7.23) | 30.57 (7.28) |
| **Contraception, N (%)** |  |  |  |  |  |
| Yes | 100 (44.84) | 78 (45.88) | 100 (46.08) | 36 (11.61) | 36 (12.29) |
| No | 123 (55.16) | 92 (54.12) | 117 (53.92) | 274 (88.39) | 257 (87.71) |
| **Strong Pain Killer, N (%)** |  |  |  |  |  |
| Yes | 183 (82.06) | 144 (84.71) | 177 (81.57) | 216 (69.68) | 203 (69.28) |
| No | 40 (17.94) | 26 (15.29) | 40 (18.43) | 94 (30.32) | 90 (30.72) |
| **Number of live births, N (%)** |  |  |  |  |  |
| 0 | 58 (26.01) | 38 (22.35) | 54 (24.88) | 133 (42.90) | 124 (42.32) |
| 1 | 54 (24.22) | 48 (28.24) | 54 (24.88) | 51 (16.45) | 47 (16.04) |
| 2+ | 111 (49.78) | 84 (49.41) | 109 (50.23) | 126 (40.65) | 122 (41.64) |
| **Depression/anxiety, N (%)** |  |  |  |  |  |
| None | 102 (45.74) | 78 (45.88) | 98 (45.16) | 166 (53.55) | 157 (53.58) |
| Moderate | 103 (46.19) | 84 (49.41) | 101 (46.54) | 123 (39.68) | 118 (40.27) |
| Extreme | 18 (8.07) | 8 (4.71) | 18 (8.29) | 21 (6.77) | 18 (6.14) |
| **Alcohol Use (units/wk)** |  |  |  |  |  |
| Mean (SD) | 3.89 (6.65) | 3.68 (7.08) | 3.91 (6.71) | - | - |
| **Two or more miscarriage/stillbirth, N (%)** |  |  |  |  |  |
| Yes | 30 (13.45) | 22 (12.94) | 28 (12.90) | - | - |
| No | 193 (86.55) | 148 (87.06) | 189 (87.10) | - | - |
| **Blood in stool, N (%)** |  |  |  |  |  |
| Yes | 49 (21.97) | 36 (21.18) | 49 (22.58) | - | - |
| No | 174 (78.03)) | 134 (78.82)) | 168 (77.42)) | - | - |
| **Model baseline pain score, Mean (SD)** | 6.80 (2.88) | 5.91 (3.38) | 5.45 (3.08) | 6.79 (2.46) | 5.71 (3.05) |

| **Table A5: Additional analyses - Developed model odds ratios, transformations, and performance statistics** | | | | | | |
| --- | --- | --- | --- | --- | --- | --- |
|  | **Models for External Validation** | |  | **Models Considering Full List of Candidate Factors (Internally Validated Only)** | | |
|  | **Dysmennorhea model** | **Dyspareunia model** |  | **Dysmennorhea model** | **Dyspareunia model** | **Non-cyclic pelvic pain model** |
| number of obs. | 223 | 170 |  | 205 | 158 | 199 |
| number of pain change events (%) | 76 (34.1) | 62 (36.5) |  | 70 (34.2) | 56 (35.4) | 80 (40.2) |
|  |  |  |  |  |  |  |
| baseline pain score, OR (95% CI) | 0.76 (0.61-0.96) | 0.96 (0.93-0.99) |  | 0.71 (0.55-0.92) | 0.95 (0.92-0.99) | 1.38 (1.21-1.57) |
|  |  |  |  |  |  |  |
| Alcohol use (units/wk), OR (95% CI) | No records in LUNA | |  | 1.04 (0.99-1.11) | - | 1.06 (0.99-1.12) |
|  |  |  |  |  |  |  |
| Use of hormonal contraception, OR (95% CI) | 0.46 (0.25-0.84) | - |  | 0.47 (0.25-0.89) | - | 0.58 (0.30-1.13) |
|  |  |  |  |  |  |  |
| Blood in stool/rectal bleed, OR (95% CI) | No records in LUNA | |  | - | 2.98 (1.23-7.25) | 0.42 (0.17-1.02) |
|  |  |  |  |  |  |  |
| Age, OR (95% CI) | - | 3.0^-7^ (2.5^-11^,0.004) |  | - | 4.1^-8^ (1.11^-12^,0.002) | - |
|  |  |  |  |  |  |  |
| Use of strong painkillers, OR (95% CI) | - | - |  | - | 0.40 (0.13-1.23) | - |
|  |  |  |  |  |  |  |
| Two or more miscarriage/stillbirth, OR (95% CI) | No records in LUNA | |  | 1.99 (0.81-4.86) | - | - |
|  |  |  |  |  |  |  |
| No live births vs. 1, OR (95% CI) | - | - |  | - | - | - |
| No live births vs. 2+, OR (95% CI) | - | - |  | - | - | - |
|  |  |  |  |  |  |  |
| No depression/anx vs. moderate, OR (95% CI) | - | - |  | - | - | - |
| No depression/anx vs. severe, OR (95% CI) | - | - |  | - | - | - |
|  |  |  |  |  |  |  |
| INTERCEPT (SE) | -0.28 (0.21) | 0.43 (0.55) |  | -0.28 (0.21) | 0.43 (0.55) | -0.12 (0.23) |
|  |  |  |  |  |  |  |
| Age transformations | - | (age/10)^-2^ - 0.08 |  | - | (age/10)^-2^ - 0.09 | - |
| Alcohol use transformations | No records in LUNA | |  | alcohol - 3.48 | - | alcohol - 3.49 |
| Baseline pain score transformations | ((baseline + 1)/10)^-2^ - 1.64 | ((baseline+1)/10)^-2^ - 2.10 |  | ((baseline + 1)/10)^-2^ - 1.61 | ((baseline + 1)/10)^-2^ -2.04 | -1(baseline - 5.52) |
|  |  |  |  |  |  |  |
| Optimism adjusted C-statistic* | 0.610 | 0.677 |  | 0.631 | 0.705 | 0.719 |
| Optimism adjusted C-slope* | 0.700 | 0.763 |  | 0.610 | 0.699 | 0.731 |
| Optimism adjusted CITL* | 0.011 | 0.003 |  | -0.014 | -0.004 | 0.001 |
| \| *Predictor may be associated with increased odds of a pain reduction event for the model at 5% significance level (i.e. odds ratio>1.0)* \| \| --- \| | | | | | | |
| \| *Predictor does not significantly change odds of a pain reduction event for the model at 5% significance level (i.e. odds ratio crosses 1.0)* \| \| --- \| | | | | | | |
| \| *Predictor may be associated with decreased odds of a pain reduction event for the model at 5% significance level (i.e. odds ratio<1.0)* \| \| --- \| | | | | | | |
| **Odds ratios from originally developed models are presented and represent the odds of a pain reduction event occuring at six-month follow-up, but performance statistics are presented after internal validation and optimism adjustment of the original models* | | | | | | |
