# Supplementary material for: Predicting pain reduction following laparoscopic surgery for endometriosis: a retrospective cohort study using UK national and research databases
Source: BMJ Open. 2025 Aug 27;15(8):e099374. doi: 10.1136/bmjopen-2025-099374 (PMC12410623; doi:10.1136/bmjopen-2025-099374)
Supplement: Supplementary file 2 [file bmjopen-2025-099374.reviewer_comments.pdf]

PEER REVIEW HISTORY

BMJ Open publishes all reviews undertaken for accepted manuscripts. Reviewers are asked to complete a checklist review form and are provided with free text boxes to elaborate on their assessment. These free text comments are reproduced below.

ARTICLE DETAILS

Title (Provisional)

Predicting pain reduction following laparoscopic surgery for endometriosis: a retrospective cohort study using UK national and research databases

Authors

Mustard, Connor; Snell, Kym; Lee, Kim May; Pike, Cleo; Bhogal, Sharandeep; Horne, Andrew; Dodds, Julie; Allotey, John; Rivas, Carol; Ball, Elizabeth

---

VERSION 1 - REVIEW

**Reviewer**	**1**
**Name**	**La Marca , Antonio**
**Affiliation**	**University of Modena and Reggio Emilia**
**Date**	**16-Feb-2025**
**COI**	**None**

Intersting article whose main findings may have clinical implications

My major concerns are related to some points

1. the main objective of this study is to find the predictors of pain relief after surgery. Surgery for endometriosis is not perfomed in a standard way and in many cases is suboptimally performed. Typically there are some referral centers for severe cases, while mild cases may be treated in peripheral centers. The outcome (pain relief) could be strongly dependent on the ability of the surgeon that performed the operation.

2. Can be a ROC of 0.7-0.8 considered "good"?

3. As the most relevant part of this reasearch article in based on the logistic regression and on statistics, I would reccomend the Editor to send this article to a statistician for reviewing it

---

**Reviewer**	**2**
**Name**	**Restaino, Stefano**
**Affiliation**	**Udine University Press**

**Date**	**19-Feb-2025**
**COI**	**None**

Dear Authors,

I carefully read and evaluated the paper "CRESCENDO: Creating a Clinical Prediction Model to Predict Surgical Success in Endometriosis"- The principal aim of this paper is to develop and validate clinical prediction models to help predict clinically meaningful pain reduction following therapeutic laparoscopy.

I have the following considerations:

- "Introduction" section: I would specify that the gold standard is medical therapy, and surgical therapy is to be considered in the event of failure or worsening of the clinical scenario.

- "Material and methods" section:

o the criteria for inclusion and exclusion should be more accurately defined.

o In the MEDAL and LUNA study, compared to the BSGE DB used, is there no risk of overlap of patients? It should be specified

o were only laparoscopies or also robotic surgeries performed? In case there are both, I would suggest talking about minimally invasive surgery.

**Reviewer**	**3**
**Name**	**Dack, Kyle**
**Affiliation**	**University of Bristol, MRC Integrative Epidemiology Unit**
**Date**	**26-Apr-2025**
**COI**	**None**

Statistical review summary:

This is a well written paper, for the most part it is clear and transparent in the methods, and the statistical analysis used a valid approach to developing predictive models. I have a few minor suggestions to improve the clarity of the paper.

Abstract:

1. Setting: Suggest clarifying that the two external databases were used for exploratory analysis.

2. The outcomes are not consistent with those described in the methods section. In the methods: "Five clinically relevant outcomes were chosen to quantify the impact of surgery

based on previous work (5): dysmenorrhea, dyspareunia, dyschezia, non-cyclical pelvic pain (NPP), and quality of life (QoL).". Currently the abstract "Primary and secondary outcome measures" contains the statistical endpoints used to evaluate model parameterization.

If there is the space to report both of these in some condensed form it would be very helpful, because otherwise the abstract results are quite difficult to understand – on first read of the abstract I was expecting a single model of post-therapy pain reduction.

Introduction

3. No suggestions.

Methods

4. "Following development all models were internally validated." This is explained in detail in the statistical section, but since it is mentioned here, a brief explanation of how the internal validation was performed would help readers.

5. "Fewer outcomes and potential predictors were available in the dataset used for external validation (MEDAL)" In the study population section, this database and LUNA were described as being used for exploratory analysis and external validation was not mentioned. I suggest changing or adding to this that they were also used for validation.

6. Figure 1. There are three outcomes reported, but not dyschezia and QOL which were listed in the "outcomes" subheading, due to these not being in the external validation dataset. But these are still listed as outcomes and I assume internally validated? If so, shouldn't they be in the first box and just not in the second box? Or perhaps clarify in the outcome section that the three outcomes were the primary/main analysis, and the two others were secondary due to lack of external validation.

7. Model development/external validation: this is all clear and reasonable, no suggestions.

Results

8. Table A4. I suggest replacing the symbol in power statistics such as 3.0^-7 with a superscript, which should be possible in most word processors.

9. Figure A2. In the main manuscript these are low resolution, I am unsure if it is due to how the pdf is created, but if the original images were low resolution, please consider increasing.

10. Table 1. Typo in the column headings: demographic characteristics.

11. The results text reports the findings clearly, no suggestions.

Discussion

12. The font changes in certain parts – please check.

13. The results are interpreted accurately, including a thorough discussion of limitations, and I have no comments or suggestions.

VERSION 1 - AUTHOR RESPONSE

Reviewer 1 - Dr. Antonio La Marca , University of Modena and Reggio Emilia
Comments to the Author:
Intersting article whose main findings may have clinical implications
My major concerns are related to some points
1. the main objective of this study is to find the predictors of pain relief after surgery. Surgery for endometriosis is not perfomed in a standard way and in many cases is suboptimally performed. Typically there are some referral centers for severe cases, while mild cases may be treated in peripheral centers. The outcome (pain relief) could be strongly dependent on the ability of the surgeon that performed the operation.
• Agree, surgical technique and expertise vary and may influence pain relief outcomes. Our methods section notes that the BSGE dataset is derived from specialist endometriosis centres which are likely to have high surgical standards. To clarify this point we will add the follow to the Materials and Methods section under Study Population: "The BSGE maintains a regularly updated UK-wide database containing data from accredited specialist endometriosis centres, ensuring surgeries were performed by surgeons with expertise in advanced laparoscopic techniques." We will also acknowledge this limitation in the Discussion: "Secondly, differences in surgical expertise may play a role in this study. While inter-surgeon variability cannot be ruled out, our use of data from BSGE-accredited centres helps minimise variability in surgical expertise, though this variability may remain as a potential unmeasured confounder". Additionally, we will update some of our Discussion, in the Main findings section, where we highlight that these results are in line with our systematic review findings calling out that these results are likely linked to high surgical standards: "This is in line with the systematic review findings that removal of deep uterosacral endometriosis reduced all five pain types when women were treated specialist centres."
2. Can be a ROC of 0.7-0.8 considered "good"?
• Thank you for highlighting this. Upon further review a C-statistic ranging from 0.7-0.8 is commonly interpreted in predictive modelling literature as displaying "acceptable to good" discrimination. We will clarify in the Results and Discussion: ""Models with C-statistics between 0.7–0.8 are considered to demonstrate acceptable to good discrimination"
3. As the most relevant part of this research article in based on the logistic regression and on statistics, I would recommend the Editor to send this article to a statistician for reviewing it
• Thank you, we have received statistical feedback on this project.

Reviewer 2 - Dr. Stefano Restaino, Udine University Press
Comments to the Author:
Dear Authors,
I carefully read and evaluated the paper "CRESCENDO: Creating a Clinical Prediction Model to Predict Surgical Success in Endometriosis"- The principal aim of this paper is to develop and validate clinical prediction models to help predict clinically meaningful pain reduction following therapeutic laparoscopy.

I have the following considerations:
1. "Introduction" section: I would specify that the gold standard is medical therapy, and surgical therapy is to be considered in the event of failure or worsening of the clinical scenario.

• Thank you for this suggestion. We propose adding the following to the introduction: “Current gold standard treatment for endometriosis involves a combination of approaches including hormonal treatments (e.g. analgesics, drugs that suppress ovarian function), multidisciplinary care (e.g. pain management and psychological support), and/or surgery to remove the lesions (usually performed laparoscopically).”
2. Material and Methods: The criteria for inclusion and exclusion should be more accurately defined.
• Propose revising the Materials and Methods section as follows: “For model development and validation, original datasets were limited based on our inclusion / exclusion criteria. Inclusion criteria: women aged 16–65 years with laparoscopically confirmed (BSGE) or clinically suspected (MEDAL/LUNA) endometriosis, complete baseline and six-month follow-up pain scores for at least one outcome. Exclusion criteria: absence of endometriosis diagnosis, missing key predictors, or missing follow-up data on primary outcomes.”
3. Materials and Methods: In the MEDAL and LUNA study, compared to the BSGE DB used, is there no risk of overlap of patients? It should be specified
• There is no overlap between datasets. We propose adding the following to the Materials and Methods: “Additionally, there was no risk of patient overlap between datasets as they were collected in separate time periods and through different recruitment channels.”
4. Materials and Methods: Were only laparoscopies or also robotic surgeries performed? In case there are both, I would suggest talking about minimally invasive surgery.
• Only laparoscopic surgeries were included. Propose adding the following to the Materials and Methods: “For the datasets listed above, all procedures recorded in the datasets were conventional therapeutic / diagnostic laparoscopic surgeries.”

Reviewer 3 - Dr. Kyle Dack, University of Bristol
Comments to the Author:
Statistical review summary:
This is a well written paper, for the most part it is clear and transparent in the methods, and the statistical analysis used a valid approach to developing predictive models. I have a few minor suggestions to improve the clarity of the paper.

Abstract:
1. Setting: Suggest clarifying that the two external databases were used for exploratory analysis.
• We will revise the Abstract Setting section as follows: “Analysis of a UK nationwide, specialist centre, surgical database (BSGE) [2013-2019, N=9,171] and two research databases, MEDAL [2011-2013, N=667] and LUNA [1998-2005, N=592] for exploratory analyses and external validation.”
2. The outcomes are not consistent with those described in the methods section. In the methods: “Five clinically relevant outcomes were chosen to quantify the impact of surgery based on previous work (5): dysmenorrhea, dyspareunia, dyschezia, non-cyclical pelvic pain (NPP), and quality of life (QoL).”. Currently the abstract “Primary and secondary outcome measures” contains the statistical endpoints used to evaluate model parameterization.
If there is the space to report both of these in some condensed form it would be very helpful, because otherwise the abstract results are quite difficult to understand – on first read of the abstract I was expecting a single model of post-therapy pain reduction.
• Thank you for this suggestion. We propose revising the Abstract Primary and secondary outcome measures section as follows: “Following model development and internal validation, primary outcome measures included model performance statistics for discrimination (C-statistic) and calibration (calibration slope and calibration-in-the-large) for pain-improvement

models for each of the 5 clinically meaningful pain domains."

Introduction
3. No suggestions.

Methods
4. "Following development all models were internally validated." This is explained in detail in the statistical section, but since it is mentioned here, a brief explanation of how the internal validation was performed would help readers.
• We propose revising that sentence in the Materials and Methods section as follows: "Following development all models were internally validated using bootstrap resampling to assess optimism-adjusted performance."
5. "Fewer outcomes and potential predictors were available in the dataset used for external validation (MEDAL)" In the study population section, this database and LUNA were described as being used for exploratory analysis and external validation was not mentioned. I suggest changing or adding to this that they were also used for validation.
• In the Materials and Methods Study models section we highlight: "Additional exploratory analyses included developing models using predictors that were known preoperatively. Our aim was to estimate pain reduction in women who had pelvic pain/suspected endometriosis but who had not yet undergone laparoscopy. These additional models were meant to reflect a real-life clinical scenario during pre- operative counselling, when only preoperative factors are known. These models were developed using MEDAL and externally validated in LUNA (see supplementary methods)." Additionally, we propose revising the Materials and Methods Predictors sections with the following: "Potential predictors available in MEDAL (for additional exploratory model development) and LUNA (for external validation of those models) are outlined in the supplementary methods."
6. Figure 1. There are three outcomes reported, but not dyschezia and QOL which were listed in the "outcomes" subheading, due to these not being in the external validation dataset. But these are still listed as outcomes and I assume internally validated? If so, shouldn't they be in the first box and just not in the second box? Or perhaps clarify in the outcome section that the three outcomes were the primary/main analysis, and the two others were secondary due to lack of external validation.
• Thank you for this suggestion. We will update Figure 1 (and Figure A1 in the appendix) to include all models that were internally validated.
7. Model development/external validation: this is all clear and reasonable, no suggestions.

Results
8. Table A4. I suggest replacing the symbol in power statistics such as 3.0^-7 with a superscript, which should be possible in most word processors.
• We will update Table A4 with superscripts.
9. Figure A2. In the main manuscript these are low resolution, I am unsure if it is due to how the pdf is created, but if the original images were low resolution, please consider increasing.
• We will attempt to improve the resolution for Figure A2 and re-added it to the manuscript.
10. Table 1. Typo in the column headings: demographic characteristics.
• We will correct the heading to "Demographic Characteristics".
11. The results text reports the findings clearly, no suggestions.

Discussion
12. The font changes in certain parts – please check.
• Thank you we will standardize all fonts throughout the manuscript.

13. The results are interpreted accurately, including a thorough discussion of limitations, and I have no comments or suggestions.
